# Application of Inverse Liquid Chromatography for Surface Characterization of Biomaterials

**DOI:** 10.1007/s10337-016-3049-5

**Published:** 2016-02-25

**Authors:** Katarzyna Adamska, Karol Kadlec, Adam Voelkel

**Affiliations:** Institute of Chemical Technology and Engineering, Poznań University of Technology, ul. Berdychowo 4, 60-965 Poznań, Poland

**Keywords:** Inverse liquid chromatography, Biomaterials, Surface characterization, Linear free energy relationship, Hydroxyapatite

## Abstract

In the present study, a novel approach for surface characterization of ceramic biomaterials is proposed. Two ceramic biomaterials—hydroxyapatite and β-tricalcium phosphate—were examined by means of inverse liquid chromatography. The Abraham LFER model was applied for physicochemical characteristics of the surface. Different compounds, characterized by different polarity and different donor–acceptor properties of functional group, were used as test solutes. The chromatographic experiments were carried out with two compositions of the mobile phase: pure acetonitrile (MeCN) and the mixture of acetonitrile and water in 80:20 ratio (MeCN/H_2_O). Thus, the influence of mobile phase on sorption properties of hydroxyapatite and tricalcium phosphate surface was also discussed.

## Introduction

The rapid development of implantology in last decades has triggered to a great deal of research on new materials able to replace injured tissue or even a whole organ. This type of materials, called biomaterials, should exhibit biotolerant, non-toxic, non-cancerogenic and non-mutagenic properties, chemical non-reactivity, as well as similar physical and chemical properties to the living tissue. Hydroxyapatite Ca_10_(PO_4_)_6_(OH)_2_ (HA) and β-tricalcium phosphate Ca_3_(PO_4_)_2_ (β-TCP) are universally used as osseous tissue substitute applied in bone loss regeneration. These two compounds combined in a two-phase bioceramic scaffold mixed in 80/20 ratio show greater ability for bone tissue formation in comparison to HA and TCP alone [1]. Additionally, HA has been used as a chromatographic filling (stationary phase) in protein purification due to its unique sorption properties [2, 3]. The nature of HA functional groups has not been well-known until recently. The specific array of sorption properties between HA and protein indicate that there are ionized calcium and phosphate groups responsible for anion- and cation-exchange retention mechanism, respectively. However, Ishikawa et al. have suggested that HA surface possesses three kinds of P-OH group acting as adsorption sites for CO_2_, CH_3_OH, CH_3_I and H_2_O. Calcium group can also act in the hydrolyzed form of Ca-OH [4, 5].

Nowadays, there are many techniques which can be used in physicochemical characterization of ceramic biomaterials surface, including: FTIR, Raman spectroscopy, X-ray diffraction, contact angle measurements and others [6–8]. Unfortunately, all these techniques do not enable to observe the influence of the biological environment on biomaterials’ surface. Body fluids can interact with the material and change its surface properties, and likewise, biomaterial surface influences the internal system due to, e.g. adsorption and adhesion processes. Therefore, knowing the surface physicochemical characteristics of biomaterials and determining their overall reactivity appears to be useful, e.g. to design a material with desirable surface properties. The detailed characterization of the surface layer of biomaterials may be essential, e.g. in the selection of appropriate modifiers of the surface of biomaterial or assessing its ability for cell adhesion.

Inverse liquid chromatography (ILC) seems to be a very helpful tool for carrying out this type of characteristic. It is a technique for studying solid–liquid interactions, where the investigated solid material is packed in a chromatographic column and then characterized by using probes (test solutes) of known properties, which are carried by the mobile phase. Interactions between the test solute and the material affect the measured retention parameters and the shape of the chromatographic peak. In order to determine the properties of the surface layer of the stationary phase, the application of the proper mathematical relationship as well as, linking experimentally obtained parameters with physicochemical properties is required. ILC technique can be a useful tool for biomaterials characterization, because by changing the composition of the mobile phase we are able to recreate conditions similar to those present in a living organism, e.g. by applying non-standard mobile phases (e.g. simulated body fluid) for the characterization of the biomaterials in conditions similar to the real ones [9].

In such a case the behavior of surface functional groups may be similar to that in real system.

Since 1980’s the mathematical correlation of retention parameters with physicochemistry of solute–sorbent interaction has been attracting more attention. Abraham and co-workers adapted Kamlet and Taft solvatochromic methods to chromatographic analysis giving it the form of linear free energy relationship (LFER) [10]. In this mathematical relationship, the retention parameter depends on solute solvation process which has been identified and dissected into four types of solute–solvent interactions: cavity formation–dispersive interaction, dipolarity–polarizability interaction and acidity or basicity hydrogen bonding interaction. In the case of liquid chromatography, these types of interactions are observed not only between solute and stationary or mobile phases but also take place between solvent and stationary phases. All these interactions have a major impact on the observed retention parameter. One of the widely accepted symbolic representation of LFER model in the form of multiple linear regression equation was presented by Abraham:

1 $$ \log k = c + eE + sS + aA + bB + vV $$

where log *k* is the logarithm of the solute retention factor, *c* is the linear regression coefficient. The capital letters *E*, *S*, *A*, *B* and *V* corresponds to the solute descriptors independent on the mobile/stationary phase used; *E* is the excess molar refraction *S*—dipolarity/polarizability descriptors, *A* and *B* correspond to the solute hydrogen bond acidity and basicity respectively, and *V* is the McGowan volume of the solute. The lowercase letters *e*, *s*, *a*, *b*, *v* are the system parameters reflecting the difference in solute interactions with the mobile and the stationary phase. Therefore, the value of the above-mentioned parameters might be useful for describing the physicochemical properties of biomaterial surface (in a given chromatographic condition-mobile phase composition and temperature) and estimation of the surface’s potential to form various types of intermolecular interactions.

There are few procedures involving inverse liquid chromatography, usually applied to the determine physicochemical properties of commercially available stationary phases, e.g. surface excess isotherms, silanol activity and hydrophobicity or using an aromatic sulphonic acids as test compounds [11–14]. The ILC term was introduced in mid-eighties in order to indicate that the material under investigation is the solid stationary phases contained in the column. The determination of the retention time of specific solutes (non-polar, basic, acidic) allow to determine the physico-chemical properties of the stationary phase [15–17]. Other applications include the use of this technique to the characteristics of the adsorptive properties of various materials [18–20]. Results obtained by using ILC technique might be “verified” by measurements of surface polarity with microcalorimetry [21, 22].

The aim of this study was to show the ability of ILC in the determination of the physicochemical properties of ceramic biomaterials—HA and β-TCP surface. Data collected by ILC technique were used in LFER model. The knowledge of surface properties of these materials might be useful in the estimation (prediction) of their biocompatibility, stability in various conditions and potential for future applications.

## Experimental

### Materials and Instruments

The biomaterials used as the chromatographic stationary phase included hydroxyapatite (with purity of more than 90 % purchased from Fluka) and sintered powder of β-tricalcium phosphate (≥95 %) from Sigma-Aldrich. We investigated HA and TCP not as a pure “chemical compounds”, but as a biomaterial.

Solvents applied as the mobile phase were acetonitrile for HPLC (Sigma-Aldrich) and distilled water for HPLC (POCH, Poland). Heptane, dichloromethane and dioxane (POCH, Poland) were used to determine the void volume of the column. All test solutes were at least of analytical grade: ethanol, phenyl acetate, propylamine, heptane (POCH, Poland); caffeine, aniline, phenol, nitromethane, 1,4-dichlorobenzene, benzonitrile, geraniol and butylbenzene (Sigma-Aldrich); butanone and pyridine (Fluka). Test compounds used in the experiments with values of their descriptors are given in Table 1. Various test solutes were chosen to exhibit a different polarity and donor–acceptor properties, to indicate the ability to various types of interactions.

**Table 1:** Solute descriptors used for characterization of analysed biomaterials [23–25]

Compound	Descriptors				
	*E*	*S*	*A*	*B*	*V*
1,4-Dichlorobenzene	0.825	0.750	0.000	0.020	0.9612
Aniline	0.955	0.960	0.260	0.410	0.8162
Benzonitrile	0.742	1.110	0.000	0.330	0.8710
Butylbenzene	0.600	0.510	0.000	0.150	1.2800
Caffeine	1.500	1.72	0.05	1.28	1.3632
Ethanol	0.246	0.420	0.370	0.480	0.4491
Geraniol	0.513	0.630	0.390	0.660	1.4903
Heptane	0.000	0.000	0.000	0.000	1.0949
Methyl-ethyl ketone	0.166	0.700	0.000	0.510	0.6879
Nitromethane	0.313	0.950	0.060	0.310	0.4237
Phenol	0.805	0.890	0.600	0.300	0.7751
Phenyl acetate	0.661	1.130	0.000	0.540	1.073
Propylamine	0.225	0.35	0.16	0.61	0.6311
Pyridine	0.631	0.84	0.00	0.52	0.6753

The chromatographic experiments were carried out by using the liquid chromatograph Dionex Ultimate 3000 LC System equipped with refractive index detector (Shodex, Ltd. USA), chromatographic liquid pump with maximum operating work pressure 600 bar, column oven and degasser—all from Dionex (currently Thermo Scientific, USA). The stainless steel column (2.1 mm i.d × 250 mm) was purchased from Applied Research Europe, Germany.

**Fig. 1:**
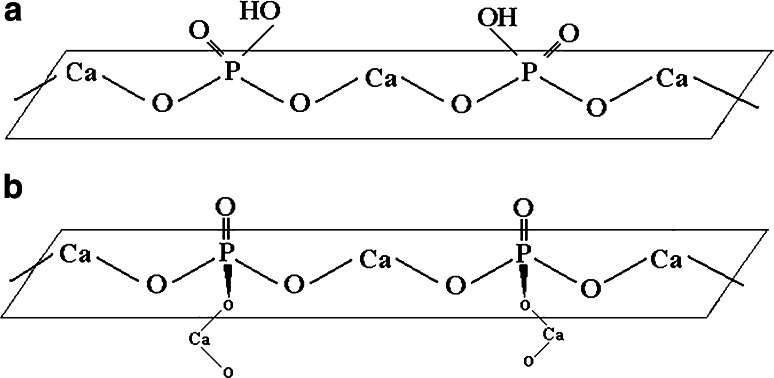
Chemical structure of HA (**a**) and β-TCP surface (**b**)

Two ceramic, bone tissue substituted biomaterials were examined by means of inverse liquid chromatography. The chemical structure of surface of examined biomaterials is presented in Fig. 1.

The non-pelletized dry powder of HA and β-TCP could not be used as the stationary phase due to mechanical deformation and high-level of compressibility that appears in chromatography column during the mobile phase flow. In such conditions, the work pressure inside the column was over 600 bar. Therefore, both biomaterials were pelletized and introduced to the stainless steel column in the form of crushed pellet. The life-time of β-TCP filling is shorter than HA and lasts about 60–70 ILC experiments, respectively. Full ILC experiment means here the collection of all test solute retention data for a given material with the use of one mobile phase.

### Column Preparation and Chromatographic Analysis

The chromatography column was filled up with one of the two biomaterials (HA or β-TCP). At first the material was pressed for 10 min with the force of 40 MPa to the form pellet. After pelletizing, the pellets were crushed in the laboratory mortar and then sieved. The fraction containing a particle in range 100–150 μm was collected and introduced to the stainless steel column. The size of the filling particles is much larger than in standard analytical approaches. The efficiency of such column is much lower (*N* = 107). However, the aim of this investigation is not the separation of the mixture of components but physicochemical characterization of material which IS NOT HPLC stationary phase. The chromatographic peak was broadened but almost satisfactory (skewness in the range of 1.01–1.10).

All test compounds were dissolved in the mobile phase at concentration of 10 mg/mL. The injection volume was 5 μm and the chromatographic conditions were as follows: flow rate—300 μL/min, column oven temperature—27 °C; the data was recorded by Chromeleon ver. 6.80. Measurements were carried out by using two mobile phases: acetonitrile 100 % (MeCN) and acetonitrile/water 80/20 (v/v) (MeCN/H_2_O).

Each sample was injected at least four times and then Q-Dixon test was applied to eliminate the results exceeding 5 % error. The average value of retention times was used to calculate the logarithm of retention factor needed to determine the log *k* parameter from Abraham equation. The LFER coefficients were calculated using multiple linear regression (Microsoft Excel).

The void volume of the filled column was determined by the pycnometric method described in detail by Rustamov et al. [26]. In order to determine void volume *V*_0_, four different solvents of differing densities were used: acetonitrile, 1,4-dioxane, heptane and dichloromethane. The void volume was calculated from the following equation:

2 $$ V_{0} = \left( {w_{1} - w_{2} } \right)/\left( {d_{1} - d_{2} } \right) $$

where *w*_1_ and *w*_2_ are the mass of the column filled with solvent with different densities *d*_1_ and *d*_2_. Retention factor is defined as:

3 $$ k = \left( {t_{R} - t_{0} } \right)/t_{0} $$

where: *k* is the retention factor, $t_{R}$—retention times, $t_{0}$—column dead- time.

## Results and Discussion

The main purpose of this work was to estimate the properties of hydroxyapatite and β-tricalcium phosphate surface described by *e*, *s*, *a*, *b*, *v* parameters of Abraham LFER model by using inverse liquid chromatography technique. Many papers in the Literature report the characterization of numerous standard chromatographic stationary phases using ILC. Various descriptors, such as e.g.: Van der Waals volume, connectivity index, partition coefficient, correlation factor, polarizability have been proposed the correlate the retention time, measured for specific probes to physico-chemical surface properties of the solid materials under investigation [15–17], but the number of papers dealing with application of ILC to characterization of ceramic biomaterials is limited.

In most cases, the authors described the properties of standard stationary phases and their ability to adsorb various compounds (aniline, phenols, sulphonic acids) [11, 12, 21, 22, 27].

Appropriate surface chemistry, but also mechanical and topographic properties are important factors in implant materials which are responsible for their biocompatibility and biological response. A variety of techniques have been proposed to characterize surface properties of biomaterials, which affect crucial processes occurring after implantation, i.e. protein adsorption and cell adhesion. Most studies rely on the use of e.g. FTIR spectroscopy to identify the chemical composition of materials [28], microscopic techniques (SEM, TEM) for surface morphology studies or analysis by X-ray diffraction [29], contact angle measurement for surface energy and wettability estimation [30], surface roughness [31]. It was found that cellular behavior largely depends on the physical and chemical characteristics of materials surface, such as topography, particle size, crystallinity and chemical composition. All these parameters (and others) also influence the protein adsorption—the initial event in biomaterial/host interaction, which is regarded as a complex process. Taking into account a chemical composition of biomaterial, it dictates the types of bonds between protein and biomaterial surface. Calcium phosphate ceramics have been widely used for applications in bone grafting and orthopedics, thus the examination of their interactions with the biological environment is a crucial issue in biomaterials research. Therefore, academic literature provides information about the role of topography [32], crystallinity [33], surface energy [34] of materials influences the main processes occurring after implantation i.e. protein adsorption and cell adhesion. However, there is still no method enabling to obtain more detailed insight in the capacity of biomaterials for various types of interaction.

The technique proposed (ILC) represents a novel technique for detailed characterization of biomaterials surface. It seems to be of significance that such information would allow the understanding of the processes, factors or mechanisms affecting biomaterials biocompatibility.

Inverse liquid chromatography enables evaluation of multi-parameter description of surface properties and thereby providing data on the capacity of a surface for different types of interactions, which seems to be useful in predicting phenomena occurring after implantation. Experimentally obtained retention data were presented in Tables 2, 3, 4 and 5.

**Table 2:** Retention times and retentions factor of test solutes put in increasing order: stationary phase—HA; mobile phase—MeCN

Compound	*t*_*R*_ (min)	*k*
Heptane	1.926	0.021 ± 0.019
Butylbenzene	2.025	0.073 ± 0.022
Phenyl acetate	2.065	0.094 ± 0.006
1,4-Dichlorobenzene	2.082	0.103 ± 0.012
Nitromethane	2.092	0.108 ± 0.028
Pyridine	2.125	0.126 ± 0.013
Benzonitrile	2.133	0.130 ± 0.004
Ethanol	2.136	0.132 ± 0.017
Caffeine	2.145	0.137 ± 0.019
Propylamine	2.158	0.143 ± 0.008
Butanone	2.204	0.168 ± 0.007
Phenol	3.138	0.663 ± 0.020
Aniline	3.543	0.878 ± 0.011
Geraniol	3.688	0.954 ± 0.031

**Table 3:** Retention times and retention factors of test solutes put in increasing order: stationary phase—β-TCP; mobile phase—MeCN

Compound	*t*_*R*_ (min)	*k*
Butanone	1.710	0.220 ± 0.021
Heptane	1.719	0.226 ± 0.033
Nitromethane	1.733	0.235 ± 0.013
Butylbenzene	1.742	0.242 ± 0.010
Phenyl acetate	1.757	0.253 ± 0.008
Aniline	1.768	0.261 ± 0.007
1,4-Dichlorobenzene	1.771	0.263 ± 0.016
Benzonitrile	1.773	0.265 ± 0.014
Pyridine	1.790	0.276 ± 0.015
Caffeine	1.795	0.280 ± 0.011
Phenol	1.823	0.300 ± 0.011
Ethanol	1.866	0.331 ± 0.025
Geraniol	1.992	0.421 ± 0.012
Propylamine	2.457	0.753 ± 0.065

**Table 4:** Retention times and retention factors of test solutes put in increasing order obtained: stationary phase—HA; mobile phase—MeCN/H_2_O

Compound	*t*_*R*_ (min)	*k*
Benzonitrile	1.877	0.115 ± 0.021
Geraniol	1.908	0.134 ± 0.008
1,4-Dichlorobenzene	1.986	0.180 ± 0.075
Phenol	2.813	0.672 ± 0.088
Butanone	3.011	0.789 ± 0.167
Aniline	3.205	0.904 ± 0.064
Nitromethane	3.251	0.932 ± 0.331
Ethanol	3.409	1.026 ± 0.045
Phenyl acetate	3.414	1.028 ± 0.184
Butylbenzene	3.550	1.110 ± 0.102
Pyridine	3.625	1.154 ± 0.162
Propylamine	3.669	1.180 ± 0.102
Caffeine	3.829	1.275 ± 0.069
Heptane	4.321	1.567 ± 0.376

**Table 5:** Retention times and retention factors of test solutes put in increase order: stationary phase—β-TCP; mobile phase—MeCN/H_2_O

Compound	*t*_*R*_ (min)	*k*
Phenyl acetate	1.398	−0.063 ± 0.017
Butylbenzene	1.474	−0.012 ± 0.002
Propylamine	1.655	0.109 ± 0.011
Butanone	1.712	0.147 ± 0.013
Geraniol	1.734	0.162 ± 0.024
Nitromethane	1.750	0.173 ± 0.002
Heptane	1.751	0.173 ± 0.004
Ethanol	1.782	0.194 ± 0.006
Benzonitrile	1.822	0.221 ± 0.007
Phenol	1.848	0.238 ± 0.003
1,4-Dichlorobenzene	1.851	0.240 ± 0.012
Aniline	1.855	0.243 ± 0.004
Caffeine	1.885	0.263 ± 0.009
Pyridine	1.886	0.264 ± 0.002

In Table 5, the values of retention factor *k* for the two first probes are negative. However, in some cases the retention time might be lower than dead time. According to definition, the dead time of chromatographic column is determined by the compound which is not retained by the stationary phase, but is able to get into the all pores within the adsorbent particles. But if the unretained compound cannot get into the pores (e.g. due to its high molecular mass), the retention time of such compound will be lower than dead time. This type of situation is sometimes observed in gel permeation chromatography, where the high molecular weight compounds are too big to be able to get into the pores of lower diameters. Compounds with negative values of k have not been taken into account in LFER model calculation. Despite on this inaccuracy of *V*_0_ measurements, the differences in retention times of test solute was obtained which was necessary to characterization of biomaterials surface by LFER model. The *e*, *s*, *a*, *b*, *v* parameters, on their own, do not have any physicochemical sense (apart from negative and positive value only). The physicochemical characterization consist on comparison of the values of these parameters, obtained from different chromatographic modes.

**Fig. 2:**
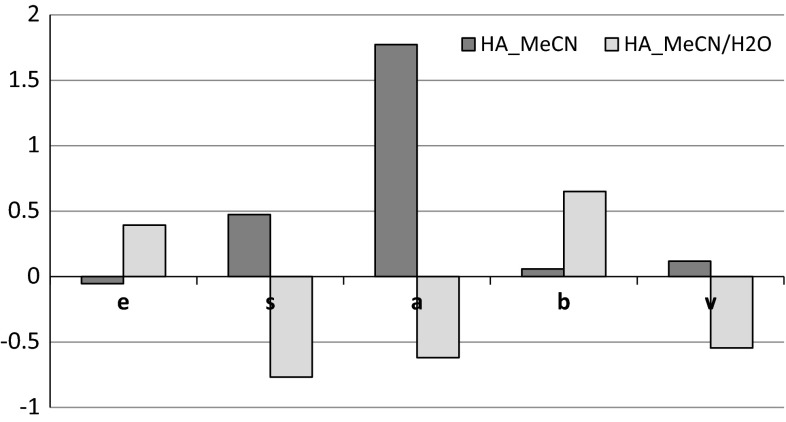
Influence of the mobile phase for *e*, *s*, *a*, *b*, *v* obtained for HA surface

**Fig. 3:**
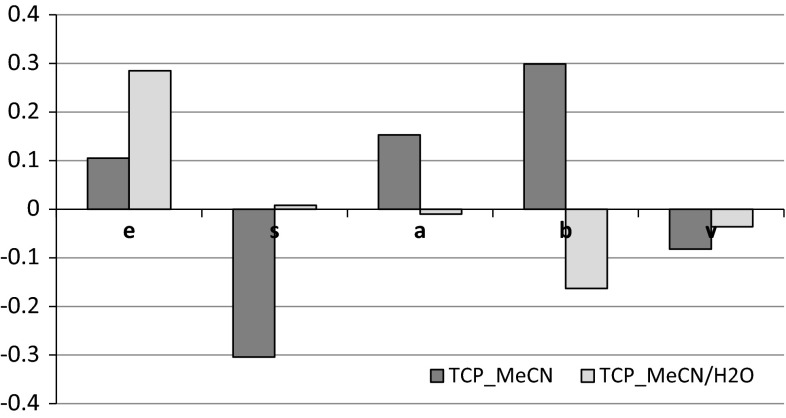
Influence of the mobile phase for *e*, *s*, *a*, *b*, *v* obtained for β-TCP surface

Figures 2 and 3 show the calculated *e*, *s*, *a*, *b*, *v* values depending upon the mobile phase used. It can be observed that hydroxyapatite and β-tricalcium phosphate have different properties and capacity for intermolecular interactions. According to Ref. [27], a positive value of a given parameter means that this type of interaction is more favorable for the stationary phase and leads to an increase in retention. On the other hand, when a given type of interaction is dominant in the mobile phase, the value is negative. Therefore we can also assume that a negative value indicates a lower capacity of the surface for a given type of interaction. The main difference in the composition of the examined ceramic biomaterials is the presence of hydroxyl groups in the hydroxyapatite’s molecule, which can influences the difference in surface’s properties of these two materials.

In Fig. 2, the parameters (*e*; *s*; *a*; *b*; *v*) determined for HA material are in −0.75/+1.75 range. However, in Fig. 3, these parameters determined for TCP material are in −0.3/+0.3 range. According to Ref. [35], in liquid–liquid partitioning LSERs reflect differences in the two phases being studied. Large coefficients reflect big differences in the solvents, whereas small or statistically insignificant coefficients means there is little or no difference between the phases with regards to the interaction ability being probed. Therefore, higher values of LSER parameter, obtained for hydroxyapatite may be correlated with higher difference of the interactions in the test compound/examined material/mobile phase system, then the respective system with tricalcium phosphate.

Taking into consideration acetonitrile as a mobile phase, only *e* coefficient appears to be slightly negative for hydroxyapatite, while the others are positive. This indicates that acetonitrile has higher propensity to interact through electron pairs than HA. However, HA is involved in dipole–dipole (*s*) and hydrogen bonding (*a* and *b*) interactions. Such behavior is related to OH group and O atoms, accumulated on HA surface, which are responsible for high degree of polarity. The only negative value of *e* descriptor might be explained by intramolecular hydrogen bond between the hydrogen from hydroxyl group and the oxygen atom both linked to the same atom of phosphorus (see Fig. 1a). This type of interactions is responsible for a significant decrease of polarizability due to excess electrons in HA functional group. A positive value of *v* indicates that the test solute will preferentially transfer from the mobile phase to the stationary phase. Small *v* value (0.118) obtained for hydroxyapatite with acetonitrile as the mobile phase indicates, that only in this system solute’s size will have a slight effect on the retention factor.

The highest value of *a* coefficient indicates higher basicity of hydroxyapatite surface in the presence of acetonitrile, which should reflect stronger interactions with hydrogen-bond donor solutes (Table 2).

It is worth noticing the relatively high value of *k* coefficient for aniline. Aniline cannot be treated as a hydrogen donor but its behavior might be explained by strong interactions with the hydrogen of OH group [36].

In the case of TCP, positive values are noted for *e*, *a* and *b* parameters, where contribution to *π* and non-bonding electrons to the dispersive interactions (*e*) and basic character of this material (*a*) are close, while its acidic properties (*b*) are little higher.

According to such assumptions, the capacity of β-TCP for interactions with basic solutes should be stronger. It is confirmed by retention data—three of the most retained compounds on β-TCP (propylamine, geraniol and ethanol) have a high capacity to form basic H-bond (Table 3). This type of interactions occurs between solute basic active centre and ligands with basic properties. Additionally, the hydroxyl group of geraniol, ethanol and phenol reveals a high affinity to the functional group gathered on β-TCP surface. All these effects affect the “balance” between the stationary phase, the mobile phase and the test solute and are reflected in the values of equation coefficients.

The addition of water causes a change in the surface physicochemical properties of the examined biomaterials. The increase of *e* value, both for HA and β-TCP is observed. The molecules of water solvate the functional polar groups of examined biomaterials, thereby affecting the increase of their excess molar refraction due to the water’s excess electrons, coming from O–H bond and excess electron from oxygen last electron shell. The highest value of *e* is for the HA and MeCN/H_2_O mobile phase composition. The HA surface possesses more polar functional groups than β-TCP, so the excess molar refraction of surface ligands, being the result of their solvation by water molecules, has to be much higher. Furthermore, solvation process influences the decrease of value of *s* parameters. The region on the HA surface functional group demonstrating electron-donor properties is either the oxygen linked to the phosphor by a double bond or in the hydroxyl group. After addition of water to the mobile phase this region is solvated, covered by H_2_O and the loss of ligand capacity to form basic H-bond occurs (negative *a* value). The highest *b* value for HA and MeCN/H_2_O might be caused by spatial arrangement of water solvating the functional groups where one of the water’s hydrogen atoms create the hydrogen bond with the oxygen atom on HA surface, while the other one is responsible for forming an H-bond with the test solute (–O–H–O–H–R). Positive *e* and *b* values should indicate stronger interactions of hydroxyapatite surface with polar solutes, what is confirmed by *k* values for the strongest retained solutes—propylamine, caffeine (Table 4). High *k* value for heptane might be explained by significant deactivation of the stationary phase by water molecules.

In the case of β-TCP, water in the mobile phase causes a strong interaction with polar compounds (*s*) reaching a high value of excess molar refraction (*e*), which corresponds with the data presented in Table 5.

The decrease of retention factors for HA stationary after addition of water to mobile phase can be explained, at least partially, by the HILIC mechanism, i.e. formation water rich phase near the surface of examined material. It cause the increase of *k* factor for polar test solutes. However, in the case of examination of β-TCP the formation of water rich phase near its surface is difficult due to the absence of OH group onto the β-TCP surface. It means that near β-TCP surface exists “water deficient” layer while in the “bulk” of mobile phase the concentration of water is higher. It may probably call it “water concentration gradient” directed from the surface to bulk region. Molecules of polar test solutes are directed to the mobile phase and their *k* factors decrease. The HA surface is more polar than β-TCP due to the presence of hydroxyl groups and, thereby, reveals much greater affinity to water than to more hydrophobic acetonitrile. Consequently, on HA surface the thin layer of absorbed water can appear. This water may interact with test solute and the retention may be a result of partition phenomenon, not a adsorption. As a result the increase of *k* factor for polar test solute might be observed. This phenomenon appears during chromatographic process in HILIC technique.

It should be noted that retention data of test solutes reflects the difference in properties of HA and β-TCP surface after water addition. Additional polar OH group originating from the water adsorbed on the polar HA surface causes stronger interaction of a solute with this surface, in contrast to β-TCP which has no OH group in its structure. Therefore, higher retention times or retention factors data for test solutes on HA may reflect the interaction of polar solutes with OH group present both in surface layer and water molecules, while for β-TCP only in water.

## Conclusions

The obtained results indicate that inverse liquid chromatography can be used as a technique for biomaterials surface characterization. Despite low efficiency of biomaterials stationary phases, the differences in retention factors between the injected test solutes are observed. Consequently, *e*, *s*, *a*, *b*, *v* LFER parameters can be calculated. One of the main problem was to collect the appropriate test compounds having the specific set of physicochemical properties. In this study, fourteen compounds had been chosen as test solutes for surface characterization. They reveal differences in value of test solutes descriptors which enable to observe different types of interactions in the system surface of the examined material, solute active centers and mobile phase.

Due to the differences in chemical structures of HA and β-TCP surface, they show capacity to act in different types of interactions which is confirmed by the calculated Abraham parameters. Two types of mobile phase were applied to observe the influence of eluent for surface properties. The addition of water caused the change in sorption properties of both examined materials, mainly decreasing their polarity. Higher retention factors in HA stationary phase may indicate that the hydroxyl group of hydroxyapatite is mainly responsible for retention mechanism. This (hydroxyl) group in non-aqueous system shows the high capacity to form basic H-bond (relatively high value of *a* coefficient for HA).
